# Emerging and Re-Emerging Diseases

**DOI:** 10.3390/pathogens10070827

**Published:** 2021-06-30

**Authors:** Wen-Hung Wang, Arunee Thitithanyanont, Aspiro Nayim Urbina, Sheng-Fan Wang

**Affiliations:** 1 Center for Tropical Medicine and Infectious Disease, Kaohsiung Medical University, Kaohsiung 80708, Taiwan; bole0918@gmail.com (W.-H.W.); aspiro.urbina@hotmail.com (A.N.U.); 2 Division of Infectious Disease, Department of Internal Medicine, Kaohsiung Medical, University Hospital, Kaohsiung Medical University, Kaohsiung 80708, Taiwan; 3 Department of Microbiology, Faculty of Science, Mahidol University, Bangkok 10400, Thailand; arunee.thi@mahidol.ac.th; 4 Department of Medical Laboratory Science and Biotechnology, Kaohsiung Medical University, Kaohsiung 80708, Taiwan; 5 Department of Medical Research, Kaohsiung Medical University Hospital, Kaohsiung Medical University, Kaohsiung 80708, Taiwan

Throughout history, infectious diseases have vastly impacted human civilization. Around fifty years ago, many people believed that the age-old battle of humans against infectious disease was virtually over and that humankind were the winners [1,2]. Unfortunately, several new diseases emerged during the past decades (such as AIDS, West Nile fever, and Legionnaires’ disease) [2,3]. In addition, certain traditional diseases resurged with full effects (such as malaria, Lyme disease, and tuberculosis) [3]. Globally, infectious diseases remain the leading cause of death, therefore it is clear that humans are yet to be declared the winners in this battle against infectious diseases.

Infectious diseases have continuously affected human health and there has been a relentless appearance of various infectious disease outbreaks reported, including the plague that scourged Europe during the Middle Ages [4], yellow fever that demolished Napoleon’s forces in Haiti during the early nineteenth century [5], and the influenza that caused a pandemic with the highest fatality (around 50 million deaths) in 1918 [6,7]. In the twentieth century, by increasing public health knowledge and interventions, the burden of infectious diseases was reduced, particularly in more developed countries [8]. Furthermore, the later industrialization and urbanization brought great improvements in sanitation, house structural development (such as windows screened with netting), and vector control. These measures collectively ameliorated the transmission rates by reducing contact with particular infectious agents [8,9]. In addition, the discovery of penicillin in 1928, as well as the development of various vaccines, ushered the era of treatment and prevention in public health, significantly contributing to the eradication of certain infectious diseases [10].

After World War II, several health organizations were established, including the US Centers for Disease Control (CDC) and the World Health Organization (WHO) that led multiple campaigns to completely eradicate specific infectious diseases, such as smallpox, a highly infectious viral disease that was completely eradicated by 1980 via vaccination campaigns [11]. These campaigns and actions established optimism and confidence to combat and control various infectious diseases worldwide.

These infectious diseases mentioned above have been identified as emerging infectious diseases (EIDs) and re-emerging infectious diseases (REIDs) [12]. EIDs are diseases that have not occurred in humans before; have occurred previously but affected only small numbers of people in isolated places; or have occurred throughout human history but have only recently been recognized as distinct diseases or as a result of a new mutant strains [12]. According to US CDC, the EIDs are defined as diseases whose incidence numbers in humans have increased in the past two decades, causing public health problems either locally or internationally [13]. REIDs are defined as diseases that were once major health problems and then declined dramatically but are recently reoccurring, leading to major health complications [13]. Although most control and prevention efforts have been implemented, both EIDs and REIDs are still onset intermittently and pose threats and burdens to human health. Some recent human EIDs and REIDs are shown in Table 1.

The occurrence of EIDs and REIDs are influenced by a variety of factors, including human behavior, microbial adaptation, ecology, globalization, and public health infrastructure [14]. In addition, most of these factors could be associated with the increasing number of human populations, overcrowding in cities with poor sanitation, fast and intense international traveling, changes in the handling or processing of food, and increased exposure of humans to microbial carrying vectors and reservoirs in nature [15]. In the fourteenth century, the first protective legislation was implemented which evolved over the centuries into the current International Health Regulations [16,17]. The first recorded quarantine regulations were reported in Venice in 1377, which was imposed to protect against diseases carried by ship borne rats [18,19]. Almost five centuries later, the first International Sanitary Conference was held which laid down certain principles for protection against disease, until 1951 when the International Sanitary Regulations was established, providing a framework on quarantine measures [20].

Furthermore, as one of the major factors, travel has always been a channel to the spreading of disease across the world. Therefore, when there are incidences of EIDs or REIDs, most countries will tighten their borders, restricting immigration and initiating strict surveillance on the products confiscated at airports and ports from travelers. For example, COVID-19, an EID, has disseminated from the first reported city (Wuhan, China) to many countries worldwide via global traveling and immigration [21,22,23]. In addition, the African swine fever (ASF), a highly contagious hemorrhagic viral disease associated with high fatality rates in domestic and wild pigs, recently caused outbreaks near the borders of China and has a remarkable capacity for transboundary and transcontinental spread potential, subsequently posing a threat to neighboring countries of China [24,25,26].

Unfortunately, certain pathogens or microorganisms related to EIDs or REIDs are being used as bioweapons to cause the threat of bioterrorism via release of such viruses, bacteria, or other agents leading to additional complexities in how EIDs or REIDs affect global health [27,28]. A number of recent bioterrorism events have had a dramatic impact on public health policies and resource allocations. Some bioterrorism agents used to cause attacks include anthrax, smallpox, melioidosis, and glanders [27]. Although advanced research and technology have provided better diagnostic capabilities as well as basic general knowledge to dissect the various factors and determinants regarding pathogenesis, virulence, cytotoxicity, and micro-host interaction, much of them still remain unknown or not yet fully understood, mandating the necessity of more efforts to address these pathogenic factors and finetune mechanisms [29].

Regarding the transmission route, several EIDs have resulted from animal-to-human transmission. The transmission of a pathogen between animals and humans is known as zoonotic transmission or zoonosis [30]. Zoonosis is currently recognized as one of the most vital pathways for the emergence of new infectious disease to humans. Reports indicated that an estimated 75% of all known EIDs originate from some type of animal reservoir [31,32]. Some examples of zoonotic disease include HIV/AIDS, SARS, MERS, several hemorrhagic fevers, Lyme disease, plague, and avian or swine origin influenza [32,33,34,35,36,37]. Each of these diseases possesses a unique etiology and a direct or indirect transmission route. Humans have a high chance to become infected due to their close contact with asymptomatic or sick animals carrying the new pathogen. Additionally, effective control of zoonotic diseases is particularly difficult, since recognizing an emerging zoonotic disease often does not occur until a major outbreak is already ongoing [31]. If the responsible zoonotic pathogen is highly transmissible, then future epidemic spread will occur even after recognition. As previously mentioned, population growth has had a drastic impact on human health. Greater zoonotic disease potential can result from this growth primarily due to an increased interaction between humans and animal habitats [38].

Several reports indicate that ecology or etiology of EIDs or REIDs are very complex, requiring a sophisticated, interdisciplinary response to reduce disease impact. One strategy to address these complexities has been coined ‘One Health’ [39,40]. One Health is an approach for designing and implementing programs, policies, legislation, and research in which multiple sectors communicate and work together to achieve better public health outcomes. One Health is also a moniker for the interdisciplinary strategy, bringing together human, animal, and environmental health professionals to address complex global health problems [41]. Overall, One Health is an important approach to improve the effectiveness of public health response and interventions as well as recruitment and application of multiple areas of expertise to work together to fight against EIDs or REIDs.

## Figures and Tables

**Table 1:** Examples of recent human emerging or re-emerging infectious disease.

Infectious Disease	Pathogen	Emerging/Reemerging	Primary Transmission
***Virus***			
West Nile fever	West Nile virus	Reemerging	Vector-borne
Hantavirus pulmonary syndrome	Hantavirus	Emerging	Zoonotic
Dengue fever	Dengue virus	Reemerging	Vector-borne
Zika virus disease	Zika virus	Reemerging	Vector-borne
Yellow fever	Yellow fever virus	Reemerging	Vector-borne
Japanese encephalitis	Japanese encephalitis virus	Reemerging	Vector-borne
Marburg hemorrhagic fever	Marburg virus	Reemerging	Zoonotic
Rift Valley fever	Rift Valley fever virus	Reemerging	Vector-borne
Ebola hemorrhagic fever	Ebola virus	Reemerging	Zoonotic
Lassa fever	Lassa virus	Reemerging	Zoonotic
Hendra virus infection	Hendra virus	Emerging	Zoonotic
Nipah virus infection	Nipah virus	Emerging	Zoonotic
Highly pathogenic avian influenza	H5N1, H7N9 influenza virus	Emerging	Zoonotic
Severe acute respiratory syndrome	SARS-CoV-1	Emerging	Respiratory (person-to-person)
Middle East Respiratory Syndrome	MERS-CoV	Emerging	Zoonotic
COVID-19	SARS-CoV-2	Emerging	Respiratory (person-to-person)
2009 Pandemic influenza	Swine-origin H1N1 influenza virus	Emerging	Respiratory (person-to-person)
***Bacteria***			
Lyme disease	*Borrelia* spp.	Emerging	Vector-borne
Cholera	*Vibrio cholerae*	Reemerging	Waterborne
Plague	*Yersinia pestis*	Reemerging	Vector-borne
Bartonellosis	*Bartonella* spp.	Emerging	Zoonotic
Vancomycin-resistant *Staphylococcus aureus* infections	*Staphylococcus aureus*	Reemerging	Person-to-person
Pathogenic *Escherichia coli* infections	Pathogenic *E. coli* strains (O157:H7 & O104:H4)	Emerging	Foodborne
Diphtheria	*Corynebacterium diphtheriae*	Reemerging	Respiratory (person-to-person)
Typhoid fever	*Salmonella typhi*	Reemerging	Foodborne, waterborne
Multidrug-resistant tuberculosis infections	*Mycobacterium tuberculosis*	Reemerging	Respiratory (person-to-person)
***Fungal***			
*Cryptococcus gattii* infections	*Cryptococcus gattii*	Emerging	Environmental exposure
***Parasite***			
Cyclosporiasis infections	*Cyclospora cayetanensis*	Emerging	Foodborne, waterborne
Drug-resistant malaria	*Plasmodium* spp.	Reemerging	Vector-borne
***Protein***			
Variant Creutzfeldt–Jakob disease	Prion	Emerging	Zoonotic, foodborne
